# Genotypic background of the recipient plant is crucial for conferring RB gene mediated late blight resistance in potato

**DOI:** 10.1186/s12863-017-0490-x

**Published:** 2017-03-09

**Authors:** Rajesh K. Shandil, Swarup K. Chakrabarti, Bir Pal Singh, Sanjeev Sharma, S. Sundaresha, Surinder K. Kaushik, Arvind K. Bhatt, Nitya Nand Sharma

**Affiliations:** 1 ICAR-Central Potato Research Institute, Shimla, 171001 Himachal Pradesh India; 2 Division of Germplasm Evaluation, ICAR-National Bureau of Plant Genetic Resources, Pusa Campus, New Delhi, India; 3 Department of Biotechnology, Himachal Pradesh University, Shimla, H.P India; 4 Premas BiotechPvt. Ltd, Gurgaon, Haryana India

**Keywords:** *RB* gene, *Phytophthora infestans*, Molecular breeding, Transgenic potato

## Abstract

**Background:**

Late blight, caused by oomycetes pathogen *Phytophthora infestans* (Mont.) de Bary, is the most devastating potato disease in the world. *RB* gene from *Solanum bulbocastanum* has been shown to impart broad spectrum resistance against *P. infestans* races. In this study Katahdin transgenic event SP951 was used as male parent to cross with the popular Indian potato cultivars *viz*., Kufri Bahar (KB) and Kufri Jyoti (KJ) to enhance the late blight resistance.

**Results:**

Populations of 271 F1seedlings from the crosses KB × SP951 (87) and KJ × SP951 (184) were screened for inheritance of *RB* transgene through PCR and bioassay. Disease response based on AUDPC of different hybrid lines varied from immunity to complete susceptibility. High degree of resistance (<25% infection) was observed in KJ × SP951 derived seedlings (85.2%), whereas level of resistance in KB × SP951 (36.4% infection) derived seedlings was of low order.

**Conclusion:**

This study provides valuable genetic materials for development of potentially durable late blight resistant potato varieties. Besides, it also corroborates the fact that efficacy of R gene is not solely dependent on its presence in the variety but largely depends on the genetic background of the recipient genotype.

## Background

Potato (*Solanum tuberosum* L.) is an important horticultural food crop which has the potential to meet food demand of the fast growing human population across the world, including India. Late blight caused by the oomycetes pathogen *Phytophthora infestans* (Mont.) de Bary is the most devastating disease worldwide causing € 12 billion crop losses annually [1]. In India, late blight is more serious in temperate highlands and plateau region than in the Indo-Gangetic plains and average crop losses to the tune of 15% have been estimated which amounts to 6.7 million metric tonnes of potatoes. Integrated disease management strategy consisting of disease forecasting, fungicide spray schedules and deployment of resistant varieties has been employed to manage this disease [2].

Major R genes mainly derived from *Solanum demissum* have been exploited world over including India, to develop late blight resistant cultivars through classical breeding. The resistance conferred by these R genes, however, was short lived due to development of matching virulences of *P. infestans* in due course of time [3]. A wild diploid potato species *S. bulbocastanum* (2n = 2 × = 24) from Mexico and Guatemala, possessing very high degree of resistance to late blight controlled by *R* genes is an exception. Classical breeding approach to transfer resistance from this species to cultivated potato is not possible because of differences in ploidy and Endosperm Balance Number [4]. To overcome this problem, the *R* gene responsible for broad-spectrum resistance in *S. bulbocastanum* was cloned by two independent groups in USA (*RB*, [5]) and The Netherlands (*Rpi-blb1*, [6]). The cloning of *RB* gene opened up the possibility of using recombinant DNA technology to transfer the gene to commercially important susceptible potato varieties to diversify and strengthen late blight resistance in cultivated potatoes [7].

The *RB* gene isolated from *S. bulbocastanum* when transferred into Katahdin using *Agrobacterium tumefaciens* [5] imparted broad spectrum resistance to known races of *P. infestans* in selected transgenic events both in the greenhouse and in field experiments [7, 8]. In the present study, the *RB*-transgenic Katahdin event SP951 (obtained from University of Wisconsin, Madison, USA under USAID-ABSP II)was used as male parent in crosses with two popular Indian cultivars *viz*. Kufri Bahar (KB) and Kufri Jyoti (KJ) which occupy almost 400,000 hectares of potato area in India (CIP Social Science Working Paper 2005–06, page number 17). Kufri Jyoti is a day-neutral, widely adapted cultivar that was resistant to late blight and is grown throughout India including hills, plains and the plateau. Kufri Bahar on the other hand is susceptible to late blight but is the most preferred cultivar in west-central plains of India occupying about 60% area. In this study, introgression of *RB* gene in Indian popular potato cultivars was demonstrated for the enhancement of late blight resistance and generation of valuable genetic material for resistance breeding programme. It was also demonstrated that genotypes with variable level of late blight resistance can be developed by crossing a specific RB-transgenic event with well adapted Indian potato cultivars.

## Methods

### Plant material

The cultivar Katahdin and its transgenic event SP951 (*RB*-transgenic) used for introgression breeding was provided by University of Wisconsin, Madison, USA under USAID-ABSP II project (Agricultural Biotechnology Support Project II). The potato cultivars Kufri Bahar and Kufri Jyoti were obtained from the Division of Seed Technology, CPRI, Shimla. The population of Kufri Jyoti × SP951 (184 F1 seedlings) and Kufri Bahar × SP951 (87 F1 seedlings) found positive for *RB* gene integration, were further evaluated for late blight resistance. All the plant materials including hybrid lines were clonally propagated inside transgenic containment facility, at ICAR-CPRI, Shimla. Kufri Bahar, Kufri Jyoti, Katahdin transgenic event SP951 (*RB*-transgenic line that contains one copy of the *RB* gene, [9]), and Katahdin (untransformed) were used as controls.

### Molecular analysis of F1 seedlings

Presence of *RB* gene in the plant samples was confirmed by amplification of both N- and C-terminal regions as well as internal region of the *RB* gene sequence using three different primer pairs. The N-terminal and C-terminal portions of the gene were amplified by using the primer pairs 1–5/3–5 and cf1/cr1, respectively [8]. The allele specific primers MAMA2/INDEL-r were used to amplify the internal region of the *RB* gene [10]. The PCR reaction condition and temperature regimes as described by the respective authors were used for amplification. The RP2-f1/r5 primer pairs (collectively referred to as “RP2 primers”) were used to amplify the RNA Polymerase II subunit 2 as internal check for all the samples [11].

### DNA extraction and PCR reactions

Genomic DNA was isolated from leaves of young shoots using GenElute™ Plant Genomic DNA Mini Prep Kit (Sigma Aldrich). Template DNA from each young plant of 271 F1seedlings was extracted for PCR confirmation of *RB* gene. Multiplex PCR reaction (20 μL) was performed using 1 U of *Taq* DNA polymerase (Bangalore GeNei™, India), 100 ng of genomic DNA, 1X PCR Buffer (MgCl_2_ free), 2 mM MgCl_2_, 160 μM dNTP, 0.50 μM of each MAMA primers (MAMAF: CATCTTGAGAGAGTGAAGAATGATCT and MAMAR: CTAGTGCGCAACACAATTGAA) and 0.10 μM of each RP2 primers (RP2F: TCGTGGATTTTTCCGATCTC and RP2R: ATCTCGCTCCATCTCTCCAA) with the following amplification conditions: 94 °C for 5 min; 35 cycles comprising of 94 °C for 1 min, 55 °C for 30 s, and 72 °C for 1 min;and final extension at 72 °C for 10 min. The *RB* gene insert was also analyzed using N-terminal and C-terminal primers. PCR reaction (20 μL) was performed using 1 U of *Taq* DNA polymerase (Bangalore GeNei^TM^, India), 100 ng of genomic DNA, 1X PCR Buffer, 1.5 mM MgCl_2_, 160 μM dNTP, 0.20 μM of each N-terminal primers (RBNF: CTCATTTTACCCCTACAA and RBNR: GCGTTTTGGACCCTTTTA) and 0.20 μM of each C-terminal primers (RBCF: TAAGCATGAGTTGGAATAACT and RBCR: GCCAGTCTTCTCCTATTCCCT) with amplification conditions as mentioned above. After completion of PCR, reaction products were loaded on 1% of agarose gel (containing ethidium bromide 0.5 μg/mL) prepared in 1 X TAE buffer, and electrophoresed at 100 V for 2 h. Gel documentation was performed under UV light using a Fluor-S^TM^MultiImager (BIO-RAD).

### Southern analysis

Selected F1 lines that were PCR positive and showed late blight resistance were analyzed for the transgene integration. For this, 15 μg of genomic DNA was digested with *Hind*III (New England Biolab) which cut once in the T-DNA. The digested DNA samples were electrophoressed on a 0.8% agarose gel for 16 h. The separated fragments were transferred onto a nylon membrane (Amersham, GE Healthcare, USA) and hybridization was performed using nptII gene fragment as the hybridized probes. DNA fragments were labelled with α[32P]- dCTP using a random primer DNA labelling kit (Amersham, GE Healthcare, USA). Hybridization was performed at 65 °C for 18 h. The filter was washed at room temperature with 2× SSC and 0.1% SDS, followed by 1× SSC and 0.1% SDS, for 10 min each [12] and the image analysed using a phosphorimager (BioRad, USA).

### Late blight disease analysis of F1 seedlings

Seedlings of Kufri Bahar x SP951 (55) and Kufri Jyoti x SP951(88) positive for *RB* gene, were evaluated for foliar blight resistance. Three tubers of each clone were planted in earthen pots under transgenic containment facility. The pots were shifted to green house at 40 days after planting (DAP) and were allowed to acclimatize for six days. Thereafter, the plants were shifted to late blight screening chamber where temperature (18 ± 1 °C) and relative humidity (≥90%) were maintained. *P. infestans* isolate belonging to A2 mating type and having all virulence genes was mass cultured on tuber slices of cv. Kufri Chandramukhi. The sporangia were washed from the tuber surface in sterilized distilled water and diluted to 40,000 sporangia/ml. The sporangial suspension was incubated at 4 °C for 30 min for release of zoospores. Plants were sprayed till run off with zoospore suspension using a hand atomizer.

Disease severity was recorded at different time intervals (2, 6 & 10 days) after inoculation [13] i.e. 48, 52, 56 days after planting and populations of KB × SP951 and KJ × SP951 were grouped based on late blight resistance score (Table 1). Plants with score of > 7.0 (<25% infection) were scored as resistant whereas those with score of <7.0 (>25% infection) were scored as susceptible. Area Under the Disease Progress Curve (AUDPC) for some of the lines showing variation in degree of late blight resistance was also calculated [14].

**Table 1:** Scoring of late blight resistance based on the percentage of infected leaf tissue

Late Blight Infection (%)	>90	81–90	71–80	61–70	41–60	26–40	11–25	<10	0
Score	1	2	3	4	5	6	7	8	9

## Results

### Molecular analysis of F1seedlings

**Fig. 1:**
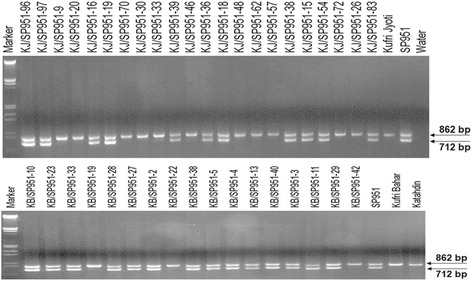
Multiplex PCR of F1seedlingsof the cross Kufri Jyoti x SP951 and Kufri Bahar x SP951using MAMA and *RP2* primers

**Fig. 2:**
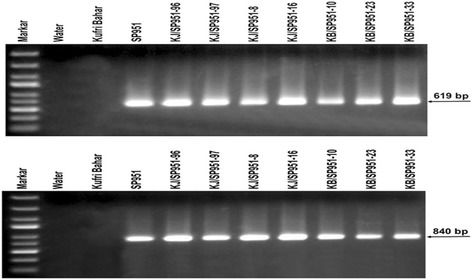
PCR confirmation of *RB* gene integration in F1seedlings using N-terminal (619 bp fragment size) and C-terminal (840 bp fragment size) primers

When Kufri Bahar and Kufri Jyoti cultivars of potato were crossed with Katahdin transgenic event SP951 (*RB*-transgenic line), 271 F1seedlings (87 of KB× SP951 and 184 of KJ × SP951) were obtained and were tested for *RB* gene integration. Stability and inheritance of *RB* gene in F1 seedlings were confirmed by PCR amplification that revealed a 712 bp fragment in 55 F1 seedlings of KB × SP951and 88 F1 seedlings of KJ × SP951 (Fig. 1) with mismatch amplification for mutation analysis (MAMA) primer. It showed that the RB gene segregated in the ratio of ~1:0.89 in the F1 which is expected in this experiment. Similarly, PCR amplification with N- and C-terminal region specific primers yielded fragment size of 619 bp (Fig. 2) and 840 bp (Fig. 2), respectively which confirmed the stability and inheritance of the transgene.

### Southern analysis

**Fig. 3:**
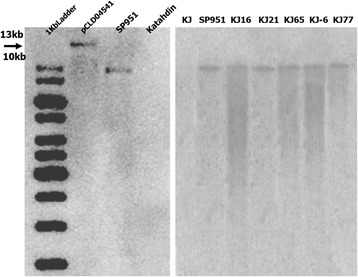
Copy number analysis of parental line SP951 and F1 hybrid lines

The integration of the transgene was confirmed by Southern hybridization in the parental line SP951 as well as in the selected F1 genotypes (Fig. 3). The result showed that the transgene copy number was the same (single copy) in the parental transgenic Katahdin SP951 as well as in the progeny lines. This confirmed stable integration of the transgene in the genome of the RB-transgenic Katahdin line SP951 and segregation of the inserted locus in the progeny of crosses involving adapted Indian potato cultivars as female parent and SP951 as male parent.

### Evaluation for foliar blight resistance

**Fig. 4:**
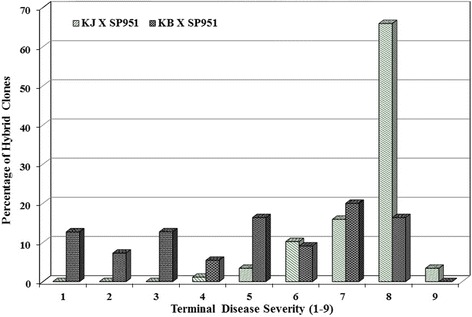
Distribution of *RB* positive population of Kufri Jyoti × SP951 and Kufri Bahar × SP951 (F1 seedlings) in the late blight resistance scale (Table 1) based on terminal disease severity at 10 DAI (days after inoculation)

*RB*-positive F1seedlings were further tested for their efficacy in terms of resistance to foliar blight under *P. infestans* inoculation. The terminal disease severity was 45% (10 DAI)in transgenic Katahdin event SP951 as against 100% in the control cultivars thereby signifying the effectiveness of *RB* gene in providing resistance to late blight. The degree of late blight resistance in F1 progenies varied from immunity to complete susceptibility (Fig. 4). The distribution of the progeny (88 of KJ × SP951 clones)for late blight disease resistance scores are as follows: 1.1% (score 4), 3.4% (score 5), 10.2% (score 6), 15.9% (score 7), 65.9% (score 8) and 3.4% (score 9). All these clones had late bight infection <70% thereby demonstrating the effectiveness of *RB* gene in imparting resistance. Few seedlings like KJ/SP951-96, KJ/SP951-97 and KJ/SP951-104 showed very high degree of resistance (score 9, Fig. 4). Contrary to this, 12.7, 7.3, 12.7, 5.5, 16.4, 9.1, 20.0 and 16.4% seedlings of KB x SP951 were scored 1, 2, 3, 4, 5, 6, 7 and 8, respectively. In total 63.6% seedlings of the cross combination were susceptible (>25% infection) to late blight (Fig. 4).

**Fig. 5:**
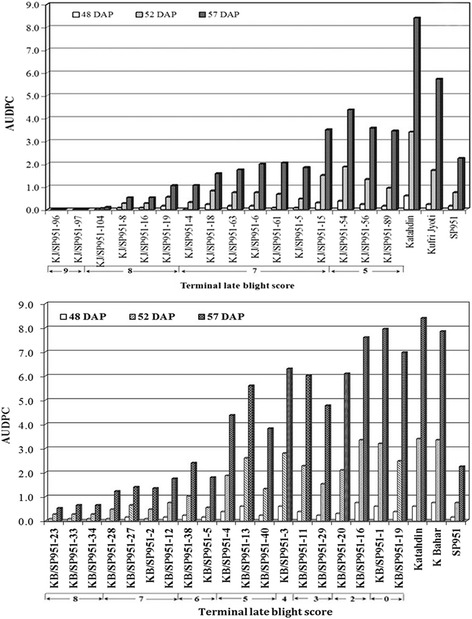
Progression of late blight infection in terms of Area Under the Disease Progress Curve (AUDPC) in Kufri Jyoti x SP951 and Kufri Bahar x SP951seedlings. The lines were grouped in the scale 1–9(DAP: Days after planting)

Late blight infection in KJ/SP951-4 at 52 DAP was 15% only which almost ceased thereafter. A very high level of late blight resistance was recorded in KJ/SP951-96, KJ/SP951-97, KJ/SP951-104, KJ/SP951-8, KJ/SP951-16 and KJ/SP951-19seedlings (Fig. 5). Whereas in KJ/SP951-4, KJ/SP951-18, KJ/SP951-63, KJ/SP951-15, and KJ/SP951-54 seedlings it was 20 to 50% at 52 DAP, the disease progression almost ceased during 52–57 DAP. On the other hand, very few seedlings of the cross KB × SP951 showed inhibitory effect on late blight progression (Fig. 5). In KB/SP951-4 (50% at 52 DAP), KB/SP951-13 (60% at 52 DAP) and KB/SP951-3 (70% at 52 DAP), late blight progression almost ceased even after very high infection percentage (50–70%) (Fig. 5).

Based on AUDPC, KB x SP951 seedlings were found more susceptible than those derived from KJ x SP951. Lowest AUDPC values (0–2) were recorded for 36 seedlings of KJ × SP951 whereas only one hybrid of KB × SP951 fell in this category. AUDPC values for Kufri Jyoti seedlings were lower (139 out of 184 hybrid lines showing AUDPC values < 1) than Kufri Bahar seedlings (10 out of 87 hybrid lines showing AUDPC values < 1) indicating higher inherent late blight resistance in Kufri Jyoti seedlings. All the KJ × SP951 seedlings had better resistance than the maternal parent Kufri Jyoti whereas seventy six (out of 184 hybrid lines)were better than the paternal parent SP951 in terms of late blight resistance. This clearly indicates effectiveness of *RB* gene in inhibiting progression of late blight infection.

## Discussion

Many tuber-bearing wild *Solanum* species are known to possess high degree of resistance to late blight. The *RB*/*Rpi-blb1* gene cloned from the *S. bulbocastanum*, when integrated into four popular potato cultivars susceptible to late blight, *viz*. Katahdin, Superior, Dark Red Norland, and Russet Burbank, conferred broad-spectrum late blight resistance consistently with little or no impact on total yield [5, 8]. The introgression of late blight resistance from wild *Solanum* species into currently cultivated potato through classical breeding is a long-term process. For instance, it took 46 years of breeding efforts to develop two varieties (Bionic and Toluca) from the first bridge cross between *S. acaule* (4x) × *S. bulbocastanum* (2x) [1]. On the other hand, development of stable transgenic potato plants with new *R* gene(s) derived from another potato species is relatively simple and has only a minor impact on the genetics of the recipient cultivated variety [8]. However, bio-efficacy of directly regenerated transgenic plants may differ because of differences in sites of gene integration, copy number variation of the inserted gene as well as genetic background of the recipient genotype(s). Therefore, the present attempt was made to develop RB-transgenic potato varieties by crossing well adapted indigenous potato cultivar(s) with well-characterized RB transgenic event of another exotic potato variety through conventional breeding.

A large variation in the level of late blight resistance in the F1population derived from KJ × SP951 and KB × SP951 was recorded. F1 seedlings derived from KJ × SP951 showed better resistance compared to those derived from KB × SP951. High throughput gene expression analysis of the selected F1 seedlings showed conflicting results with respect to level of expression of R and R-like genes vis-à-vis late blight resistance level (unpublished data). It, therefore, indicated that expression level of *RB* gene alone in particular F1 genotype was unable to explain its observed level of late blight resistance. Instead, it may be dependent on genotypic background, specifically combinations of pathogenesis related (PR) gene alleles with the RB allele in particular F1genotype derived from the introgression cross. Experiments to find out the best combination of RB gene with other gene(s) for obtaining best level of late blight resistance is underway in our laboratory. The correlation between the transcript abundance of *RB* gene and late blight resistance has been established recently [9, 15] in different transgenic events of the same variety. However, it may not be sufficient to explain the observed difference in the level of late blight resistance in the F1 progeny of a particular transgenic event as indicated in our study. Similarly, the *Sgt1* gene has been reported essential for the *RB*-mediated late blight resistance and *Sgt1*- silenced transgenic lines of potato failed to exhibit *RB*- mediated late blight resistance [16]. In some instances, pathogen induced up-regulation of *R* genes has also been noted previously in other pathosystems [17–20].

The Indian cultivar Kufri Jyoti was bred as a late blight resistant cultivar in 1968 using *S. demissum* as resistant parent. It possesses three major *R* genes (R3, R4, R7) but resistance in this cultivar was compromised due to development of matching virulences during 1980s that knocked down all the three *R* genes. However, the downstream signal transduction cascade as well as PR proteins in Kufri Jyoti was presumably very effective since it showed high level of resistance to late blight before its breakdown due to evolution of matching races in the pathogen. It was evident from the high level of resistance observed in majority of F1 progeny derived from Kufri Jyoti where an effective *R* gene (RB) was introduced. Although, the stability and inheritance of the RB gene was confirmed through Southern analysis and late blight assay, variation in the resistance level of the F1 progeny having the RB gene was probably due to shuffling of other downstream genes during meiotic recombination. On the contrary, most of the progeny derived from the cross with the susceptible cultivar Kufri Bahar showed inferior level of resistance even after having RB gene, probably because of in-effective combination of downstream genes. This indicates that allelic combination of downstream genes encoding signal transduction cascades as well as pathogenesis related proteins are very important for getting desired level of resistance in a particular genotype. Besides, several other polygenic components may be responsible for the observed variation in late blight severity among the F1seedlings. For example, the*Sgt1* gene has been reported essential for the *RB*-mediated late blight resistance and this may be another reason for the observed variation in *R* gene-mediated plant defense responses [16]. An understanding of the genetic relationship within potato hybrid lines is important to establish a broad genetic base for breeding purposes [21]. Potato germplasm with reported late blight resistance should be introgressed with *RB* for stronger and more durable resistance.

The results corroborate the hypothesis that *RB* is an signal receptor protein that triggers a cache of defence proteins to induce localized hypersensitive response thereby halting the pathogenesis at the site of invasion [22]. Level of resistance depends on the combination of effective alleles of the defence proteins present in a particular genotypic background. Our findings support the above hypothesis. The cessation of late blight progression after 40–70% infection in the KB × SP951 and KJ × SP951 population, supports the results obtained for *S. bulbocastanum* clone PT29 derived somatic hybrids and a number of backcrossed progenies where the pathogen sometimes sporulated and the disease phenotype was general suppression but not elimination of symptom development [17, 23]. Promising lines were selected on the basis of per cent infection on the last day (57 day after planting, DAP) of scoring as well as AUDPC. Area under the disease progress curve is a measure of disease development over the whole course of the epidemic. The lines showing 40% or less late blight infection at 57 DAP and AUDPC values below that of control varieties Kufri Bahar and Kufri Jyoti were selected for further evaluation.

Therefore, *RB* transgene can be used effectively to reduce foliar late blight infection in cultivated potatoes. This enhanced resistance over the generation may be ascribed to the pooling of R gene activation or re-shuffling of R genes in the background of Kufri Jyoti that imparts resistance against *Phytophthora infestans*. This RB positive F1 genotypes showing different levels of late blight resistance will be useful for identifying promising downstream genes to build the future molecular breeding activities.

## Conclusions

The study confirms that the classical *R* gene (*RB*) with NBS-LRR-CC motif from the wild potato species *Solanum bulbocastanum* confers broad spectrum resistance to potato late blight. It was also established that the RB transgene could be transferred to the F1 progeny by crossing non-transgenic locally adapted cultivar with the transgenic event of the cultivar Katahdin. In addition this study demonstrated that the level of late blight resistance varied greatly within the F1 progeny, i.e. a few F1 genotype possessing the RB transgene showed immunity to late blight while some others were completely susceptible even after possessing the RB transgene. The differential late blight response could not be explained by the variation in level of RB gene expression in the F1 progeny. This study, therefore, clearly indicated that efficacy of R gene is not solely dependent on its presence in the particular genotype but largely depends on the genetic background of the recipient genotype. It will be interesting to investigate role of other downstream genes in potato for achieving satisfactory level of resistance through the classical R gene.

## Abbreviations

AUDPC: Area under the disease progress curve
DAP: Days after planting
KB/SP951: Lines obtained from Kufri Bahar crossed with SP951
KJ/SP951: Lines obtained from Kufri Jyoti crossed with SP951
MAMA: Mismatch amplification mutation assay
PR: Pathogenesis related
RB: R gene from *Solanum bulbocastanum*
SGT: Suppressor of G2 allele of S-phase Kinase Associated Protein 1 (SKP1) gene
SP951: RB transgenic Katahdin event (Male parent)
